# The Enhancement of Acylcarnitine Metabolism by 5-Heptadecylresorcinol in Brown Adipose Tissue Contributes to Improving Glucose and Lipid Levels in Aging Male Mice

**DOI:** 10.3390/nu15214597

**Published:** 2023-10-29

**Authors:** Kuiliang Zhang, Lei Jiang, Lamei Xue, Yu Wang, Yujie Sun, Mingcong Fan, Haifeng Qian, Li Wang, Yan Li

**Affiliations:** State Key Laboratory of Food Science and Technology, School of Food Science and Technology, Jiangnan University, Wuxi 214122, China

**Keywords:** 5-heptadecylresorcinol, acylcarnitine metabolism, brown adipose tissue, aging

## Abstract

5-Heptadecylresorcinol (AR-C17), a primary biomarker of whole grain (WG) consumption, has been demonstrated to improve the thermogenic activity of aging mice. However, the intricate regulatory mechanism is not fully understood. This study conducted metabolomics analysis on young and aging mice with or without AR-C17 administration after cold exposure. The results showed that the aging mice displayed lower levels of acylcarnitine (ACar) in their plasma compared with the young mice during cold exposure, and 150 mg/kg/day of AR-C17 administration for 8 weeks could increase the plasma ACar levels of aging mice. ACar has been reported to be an essential metabolic fuel for the thermogenesis of brown adipose tissue (BAT). AR-C17 had similar effects on the ACar levels in the BAT as on the plasma of the aging mice during cold exposure. Furthermore, the aging mice had reduced ACar metabolism in the BAT, and AR-C17 could improve the ACar metabolism in the BAT of aging mice, thereby promoting the metabolic utilization of ACar by BAT. Moreover, the glucose and lipid levels of aging mice could be improved by AR-C17. This study revealed a deeper metabolic mechanism involved in the AR-C17-mediated thermogenic regulation of BAT, providing a new theoretical basis for the nutrition and health benefits of WG.

## 1. Introduction

Alkylresorcinols (ARs) are special phenolic compounds in cereals’ bran fraction and have been regarded as biomarkers of whole grain (WG) consumption [1]. ARs can be found in the plasma and adipose tissue of the human body after long-term dietary intake of WG [2]. 5-Heptadecylresorcinol (AR-C17) is the primary active homolog of ARs and a major biomarker of WG consumption. AR-C17 has numerous health benefits, including neuroprotection, anti-inflammatory, anti-cancer, anti-arteriosclerosis, anti-obesity, and anti-aging activities [3,4,5,6,7,8,9]. Therefore, AR-C17 is a promising functional ingredient in WG that promotes metabolic health in the body. WG is becoming increasingly popular in daily diets worldwide due to its high content of dietary fiber, vitamins, and various functional components, such as phytosterols, polyphenols, carotenoids, and ARs [10,11]. WG foods have been demonstrated to ameliorate various metabolic syndromes, including hyperlipidemia, hypertension, hyperglycemia, and obesity [11]. Therefore, research on the health benefits of AR-C17 may help in accurately understanding the metabolic regulation mechanisms of the body by WG.

Adipose tissues are essential for the regulation of systemic energy metabolism [12]. White adipose tissue (WAT) is the major site for energy storage and plays a crucial role in maintaining energy homeostasis through its endocrine communication [13]. WAT displays large, unilocular lipid droplets (LDs) and few mitochondria, and excessive lipid accumulation in WAT leads to obesity and related metabolic dysfunction [14]. In contrast, brown adipose tissue (BAT) possesses small, multilocular LDs and a large number of mitochondria, dissipating energy to produce heat through uncoupling protein 1 (UCP1), which is localized in the mitochondria [15]. Emerging evidence has identified beige adipose tissue, derived from WAT, as a new type of thermogenic adipose tissue. This type of tissue can be induced under specific conditions, including cold exposure, pharmacological stimulation, and dietary intervention [16]. The activation of thermogenic adipose tissues is beneficial for systemic energy metabolism and the mitigation of metabolic diseases related to obesity [17]. Dietary supplementation is a more reasonable and healthy strategy for activating thermogenic adipocytes. Several studies have shown that resveratrol, rutin, curcumin, anthocyanins, capsaicin, and phenolic acids can enhance adipocyte thermogenesis by modulating various signaling pathways [11]. Therefore, exploring dietary-derived functional ingredients as activators of thermogenic adipose is a research hotspot.

Upon cold exposure, the activated BAT utilizes large amounts of free fatty acids (FFAs) and glucose for thermogenesis and to maintain metabolic homeostasis [18]. Acylcarnitines (ACar) have been identified as a novel and essential thermogenic fuel for BAT thermogenesis [19]. During cold exposure, the activation of the β3-adrenergic receptor induced by catecholamines stimulates WAT lipolysis, releasing FFAs into the plasma [12]. The FFAs in the plasma can activate hepatic nuclear factor 4α (HNF4α), which is essential for ACar production in the liver, and serve as the substrates for ACar synthesis in the liver during cold exposure [19]. HNF4α can directly stimulate the transcriptional program of the genes involved in liver ACar metabolism, including organic cation transporter 2 (OCTN2), carnitine palmitoyltransferase 1a/b (CPT1a/b), carnitine palmitoyltransferase 2 (CPT2), and carnitine-acylcarnitine translocase (CACT) [19]. Once in the plasma, ACar is transported to the BAT during cold exposure, while uptake into the WAT and liver is blocked, and BAT can directly utilize ACar for thermogenesis [19]. After entering into the BAT, ACar can be transported into the mitochondrion through CACT. Once inside, ACar can be converted into acyl-CoAs and carnitine through CPT2. These conversions enable subsequent fatty acid β-oxidation in the mitochondrial matrix, promoting the thermogenic metabolism of brown adipocytes [20]. Therefore, BAT is the endpoint of ACar metabolism and the utilization of the body in response to cold, and the ACar metabolism in BAT is essential for the ACar utilization of the body to produce heat.

Aging is linked to a decline in metabolic functions [21]. Various metabolic disorders are accompanied by aging, including impaired glucose uptake and glycogen synthesis in skeletal muscle, excessive adipose lipogenesis, and uncontrolled hepatic gluconeogenesis [22]. Therefore, maintaining the homeostasis of glucose and lipid metabolism is important for the overall metabolic health of the body during aging. The loss of BAT activity is a significant metabolic disorder in the elderly (over 65 years old), which reduces the positive effect of BAT on metabolic health [22]. The activity and expression of UCP1 in BAT decrease during aging, and there is a phenotypic switch, where brown adipocytes convert into a white-like phenotype, that occurs with age [23]. During cold exposure, the activated sympathetic nervous system (SNS) releases norepinephrine, communicating with β3-adrenergic receptors and activating BAT thermogenesis [24]. However, the activity of SNS and the responsiveness of BAT to norepinephrine are both reduced during aging, leading to the age-associated metabolic dysfunction of the BAT [22]. As an important metabolic tissue for energy regulation, BAT plays a crucial role in maintaining energy homeostasis and has been recognized as a promising target for the treatment of metabolic disorders associated with aging, such as diabetes and obesity [25]. In our previous study, we demonstrated that AR-C17 displayed great anti-aging potential, which could improve the thermogenic capacity of BAT in aging mice by regulating the Sirt3 signaling pathway [9]. However, the intricate regulatory mechanism is not fully understood and requires further exploration. In the present study, we discovered that ACar metabolism played a role in the improvement of BAT thermogenesis in aging mice regulated by AR-C17. Furthermore, AR-C17 was found to regulate the utilization of ACar, a novel thermogenic fuel, in order to promote BAT thermogenesis in aging mice, partially contributing to the improvement of glucose and lipid levels in the aging mice.

## 2. Materials and Methods

### 2.1. Materials and Reagents

AR-C17 (purity ≥ 98%, CAS: 41442-57-3) was purchased from Macklin (Shanghai, China). Fetal Bovine Serum (Cat# 12483020), high-glucose DMEM (Cat#12100046), Penicillin-Streptomycin (Cat#15140122), and 0.25% Trypsin-EDTA (Cat#25200072) were purchased from Gibco (New York, NY, USA). TRIzol Reagent (Cat#15596018CN) was purchased from Thermo Fisher Scientific (San Diego, CA, USA).

### 2.2. Animal Experiments

Compared to female mice, male mice are larger and stronger in size, and have a greater tolerance for drugs. Additionally, male mice do not estrus, greatly reducing the uncertainty in the experiment. Therefore, the experiment was conducted only in male mice. All animal experiments were performed according to procedures approved by the Laboratory Animal Ethics Committee of Jiangnan University (JN. No. 20220615c0480930[246]) and complied with the guidelines and regulations of the Guide for the Care and Use of Laboratory Animals. All mice were kept in a specific-pathogen-free animal facility with a 12 h dark-light cycle at a constant temperature of 23 ± 2 °C and relative humidity of 55 ± 5%. All mice were given free access to a normal chow diet (AIN-93G) and water. The chow composition is shown in Table 1. The young C57BL/6J male mice, aged 12 weeks, and the aging male mice, aged 18 months, were purchased from Charles River. The mice were divided into four groups: the young control group (*n* = 5), the young mice treated with AR-C17 group (*n* = 5), the aging control group (*n* = 5), and the aging mice treated with AR-C17 group (*n* = 5). AR-C17 was dissolved in 0.5% sodium carboxymethyl cellulose (CMC-Na, CAS: 9004-32-4, Macklin, Shanghai, China) aqueous solution, and the control groups received the same volume of CMC-Na aqueous solution. AR-C17 was orally administrated to the mice by gavage at the dose of 150 mg/kg/day for 8 weeks [9]. At the end of the treatment, the mice were treated with cold exposure for 48 h. All the mice were euthanized with CO_2_, and the plasma, serum, and BAT were immediately harvested and stored at −80 °C for further analyses.

### 2.3. Metabolomics Analysis

The pretreatment of samples for pseudo-targeted metabolomics analysis was conducted following the method described in a previous study, with some modifications [26]. Briefly, 200 μL plasma and ~50 mg frozen BAT of mice were separately placed in the centrifuge tubes for extraction. Individual samples were added to 500 μL cold methanol (Cat#40064260, Sinopharm Chemical Reagent, Shanghai, China) and homogenized continuously at 4 °C; then, the mixtures were sonicated for 15 min in an ice water bath. Metabolite extracts were isolated through centrifugation at 12,000× *g* for 15 min at 4 °C, and the supernatants were separated and completely dried through lyophilization. The dried samples were re-dissolved into 20% methanol and were analyzed through LC-QTRAP 5500^+^-MS/MS (Sciex, Concord, Canada). The MetaboAnalyst 5.0 software was used for data processing. MetaboAnalyst normalization, scaling, PLSDA, and a heatmap were automatically processed in the MetaboAnalyst 5.0 software.

### 2.4. Glucose Tolerance Test (GTT) and Insulin Tolerance Test (ITT)

The blood glucose of the mice was measured after cold exposure for 48 h. GTT and ITT were conducted as described previously before cold exposure [27]. GTT was carried out on mice through intraperitoneal injection with 1 g/kg body weight of D-glucose (Cat#G5767, Sigma-Aldrich, Burlington, VT, USA) after being fasted for 16 h. For ITT, the mice were injected intraperitoneally with regular human insulin (Novolin^®^ 30R Penfill^®^, Novo Nordisk, Beijing, China) at a dose of 0.75 U/kg body weight. Blood glucose levels were measured from the tail vein at 0, 15, 30, 60 and 120 min after injection using a blood glucose meter (ACCU-CHEK Instant, Roche, Basel, Switzerland).

### 2.5. Biochemical Analysis of Serum

Serum levels of total cholesterol (TC) and triglyceride (TG) were measured according to the manufacturer’s protocols (Cat#A110-1-1 and A111-1-1, Nanjing Jiancheng Bioengineering Institute, Nanjing, China). Briefly, each serum sample was added to the well of 96-well plates and then mixed with the enzyme reagent provided in the assay kit. After incubation at 37 °C for 10 min, the absorbance of each well was detected using a Microplate reader (Thermo Fisher Scientific, San Diego, CA, USA) The serum FFA was measured using a Free fatty Acids Content Assay Kit (Cat#BC0590, Solarbio, Beijing, China). Briefly, each serum sample was treated with n-Heptane: absolute methanol: chloroform (24:1:25) and centrifugated; the supernatant was used to determine the FFA levels according to the manufacturer’s protocols.

### 2.6. Cell Culture and Treatment

The brown adipocyte cells (BAC) were extracted from stromal vascular fraction (SVF)-derived preadipocytes from the BAT of newborn C57BL/6J mice using SV40 retrovirus and cultured in dulbecco’s modified eagle medium (DMEM) supplemented with 10% fetal bovine serum and 1% penicillin/streptomycin at 37 °C with 5% CO_2_ [28]. To induce differentiation, BAC was cultured in a medium with 0.1 μg/mL insulin (Cat#I1882, Sigma-Aldrich, Burlington, VT, USA), 1 nM triiodothyronine (T3, Cat#709719, Sigma-Aldrich, Burlington, VT, USA), 500 μM 3-isobutyl-1-methylxanthine (IBMX, Cat#I5879, Sigma-Aldrich, Burlington, VT, USA), 5 μM dexamethasone (DEX, Cat#D4902, Sigma-Aldrich, Burlington, VT, USA), and 0.125 mM indomethacin (Cat#I7378, Sigma-Aldrich, Burlington, VT, USA) for 48 h; then, it was replaced with another medium supplemented with 0.1 μg/mL insulin and 1 nM T3 for 4 days. The media were refreshed every other day. The differentiated mature BAC were used for further experiments. The BAC were incubated with 20 mg/mL D-galactose (D-Gal, Cat#D810318, Macklin, Shanghai, China) for 48 h to induce cell senescence. Then, the cells were treated with or without 20 μM AR-C17 for another 24 h.

### 2.7. RNA Isolation and qRT-PCR Analysis

Total RNA was extracted from tissues and cells using Trizol reagent as described in a previous study [29]. All of the RNA were quantified using a One Drop spectrophotometer. cDNAs were synthesized using Hifair^®^ Ⅲ 1st Strand cDNA Synthesis SuperMix (Cat#11141ES60, Yeasen, Shanghai, China). qRT-PCR was performed using Hieff^®^ qPCR SYBR Green Master Mix (Cat#11203ES08, Yeasen, Shanghai, China) on a QuantStudio 3 RT-PCR system (Applied Biosystem, Foster, CA, USA). As an internal control, 18S was used to normalize the data to determine the relative expressions of the target genes using the 2^−ΔΔCt^ method. The primer sequences used for qRT-PCR are shown in Table S1. The values of RNA integrity are shown in Table S2.

### 2.8. Western Blotting

The tissue and cell samples were homogenized and extracted in RIPA lysis buffer (Cat#P0013B, Beyotime, Shanghai, China) with phosphatase and protease inhibitor cocktail (Cat#HY-K0010 and Cat#HY-K0023, Roche, Basel, Switzerland), following 10 min boiling and centrifugation to collect the supernatant for subsequent analyses. The protein concentration was determined using a BCA Protein Quantification Kit (Cat#P0010, Beyotime, Shanghai, China). Samples containing equal amounts of protein (20 μg) were loaded and separated on 10% SDS-PAGE and subsequently transferred to polyvinylidene difluoride membranes (Cat#IPVH00010, Merck Millipore, Shanghai, China). Membranes were incubated with primary antibodies overnight at 4 °C. After washing three times with TBST, the membranes were incubated with Peroxidase-AffiniPure Goat Anti-Rabbit/Mouse IgG (Cat#111-035-003 and Cat#115-035-003, Jackson ImmunoResearch, West Grove, PA, USA) at a dilution of 1:1000 for 1 h. Then, the membranes were washed another three times with TBST. Finally, the blots were visualized using an X-ray film processor (Delight, Suzhou, China). Protein band intensities were quantified in the Image J (version 1.6, NIH, Bethesda, MD, USA) software. The primary antibodies are as follows: anti-OCTN2 (1:1000, Cat#16331-1-AP, Proteintech, Wuhan, China), anti-CPT1b (1:1000, Cat#22170-1-AP, Proteintech, Wuhan, China), anti-CACT (1:1000, Cat#19363-1-AP, Proteintech, Wuhan, China), anti-CPT2 (1:1000, Cat#26555-1-AP, Proteintech, Wuhan, China), anti-β-actin (1:1000, Cat#3700, Cell Signaling Technology, Beverly, MA, USA).

### 2.9. Statistical Analysis

Data are shown as mean ± standard error mean (SEM). Student’s *t*-test was performed to assess the difference between the two groups. Statistical analysis was performed using the GraphPad Prism 8.0 software (GraphPad Software, La Jolla, CA, USA). All experiments were performed at least three times, and the representative data are shown.

## 3. Results

### 3.1. Aging Mice Display Abnormal ACar Levels in Plasma after Cold Exposure

**Figure 1:**
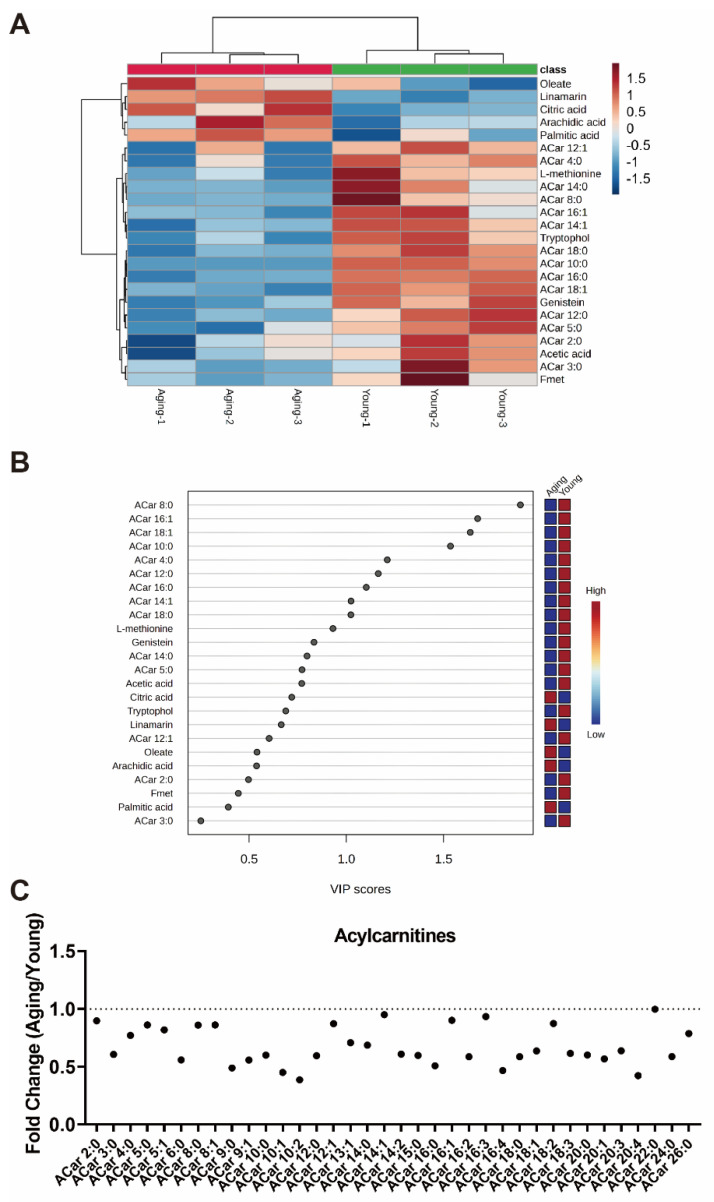
Aging mice display abnormal acylcarnitine (ACar) levels in plasma after cold exposure. (**A**) Heatmap of metabolomics analysis in plasma of young and aging mice after 48 h cold exposure (*n* = 3). (**B**) VIP score of metabolomics analysis in plasma of young and aging mice after 48 h cold exposure (*n* = 3). (**C**) Fold change of ACar levels in plasma between young and aging mice after 48 h cold exposure (*n* = 3).

To explore the metabolic regulatory mechanism underlying the decline in BAT thermogenesis associated with aging in mice, we initially conducted metabolomics analysis of the plasma samples from the young and aging mice after cold exposure. The heatmap result showed that the ACar levels in the plasma of the aging mice were significantly lower than those of the young mice during cold exposure (Figure 1A). Moreover, the VIP score indicated that ACar had a high degree of contribution to the downregulated metabolites in the plasma of the aging mice compared with the young mice during cold exposure (Figure 1B). Furthermore, the fold change results of the ACar levels in the plasma of the young and aging mice, as detected through the metabolomics, demonstrated that almost all species of ACar were downregulated in the aging mice during cold exposure (Figure 1C). The results suggested that aging mice might have a blunted response in the mobilization of ACar during cold exposure, which could contribute to the impaired cold tolerance observed in the aging mice herein [19]. Together, ACar may play a crucial role as a species of metabolites in maintaining metabolic flexibility to resist cold in aging mice.

### 3.2. AR-C17 Increases ACar Levels in Plasma and BAT of Aging Mice in Response to Cold

**Figure 2:**
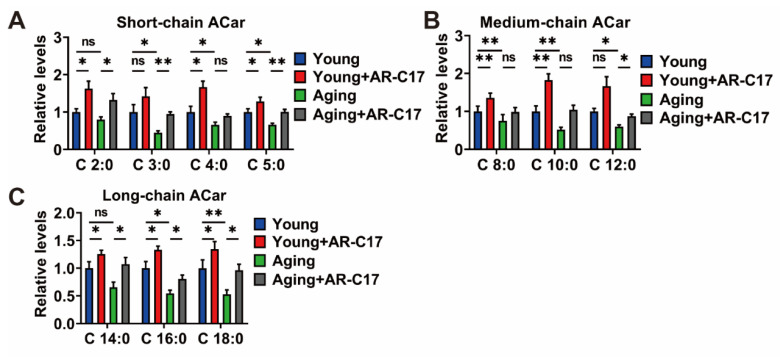
AR-C17 increases ACar levels in plasma of aging mice in response to cold. (**A**–**C**) Short-chain ACar (**A**), medium-chain ACar (**B**), and long-chain ACar (**C**) levels in plasma of young mice and aging mice treated with or without 5-heptadecylresorcinol (AR-C17) after 48 h cold exposure (*n* = 3). Data are presented as the mean ± SEM and *n* indicates the number of biologically independent experiments. Statistical significance was determined using a Two-tailed Student’s *t*-test. * *p* < 0.05; ** *p* < 0.01; ns, not statistically significant.

**Figure 3:**
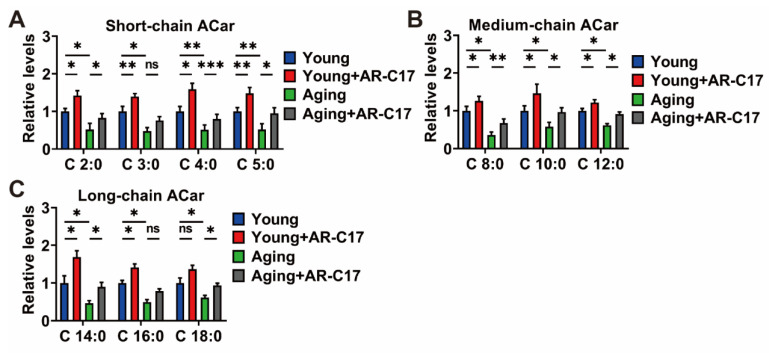
AR-C17 increases ACar levels in brown adipose tissue (BAT) of aging mice in response to cold. (**A**–**C**) Short-chain ACar (**A**), medium-chain ACar (**B**), and long-chain ACar (**C**) levels in BAT of young mice and aging mice treated with or without AR-C17 after 48 h cold exposure (*n* = 3). Data are presented as the mean ± SEM and *n* indicates the number of biologically independent experiments. Statistical significance was determined using a Two-tailed Student’s *t*-test. * *p* < 0.05; ** *p* < 0.01; *** *p* < 0.001; ns, not statistically significant.

To investigate the potential effect of AR-C17 on the ACar levels in aging mice, the levels of short-chain ACar, medium-chain ACar, and long-chain ACar in the plasma of the young and aging mice treated with or without AR-C17 after cold exposure were initially assessed. The results showed that AR-C17 could increase the ACar levels in the plasma of young mice in response to cold, and the ACar levels in the plasma of aging mice could also be increased by AR-C17 (Figure 2A–C), indicating that AR-C17 might improve the response in the mobilization of ACar in aging mice during cold exposure, thereby promoting the metabolic flexibility of ACar. Furthermore, AR-C17 increased the ACar levels in the plasma of the aging mice, which could potentially provide more thermogenic fuel for BAT thermogenesis. Next, we measured the levels of short-chain ACar, medium-chain ACar, and long-chain ACar in the BAT of the young mice and aging mice treated with or without AR-C17 after cold exposure. Our data showed that AR-C17 could increase the ACar levels in the BAT of young mice in response to cold, and the low ACar levels in the BAT of aging mice could also be increased by AR-C17 (Figure 3A–C). The results indicated that AR-C17 might promote the metabolic flux of ACar in BAT, suggesting that AR-C17 could increase the transport and utilization of ACar in BAT. However, aging mice might display a low metabolic flux of ACar in the BAT, and the transport and utilization of ACar might also decline in the BAT of aging mice. Fortunately, AR-C17 might reverse the weak ACar metabolism phenotype of the BAT in aging mice, improving the transport and utilization of ACar in the BAT and promoting BAT thermogenesis in aging mice during cold exposure.

### 3.3. AR-C17 Improves ACar Metabolism in BAT of Aging Mice in Response to Cold

**Figure 4:**
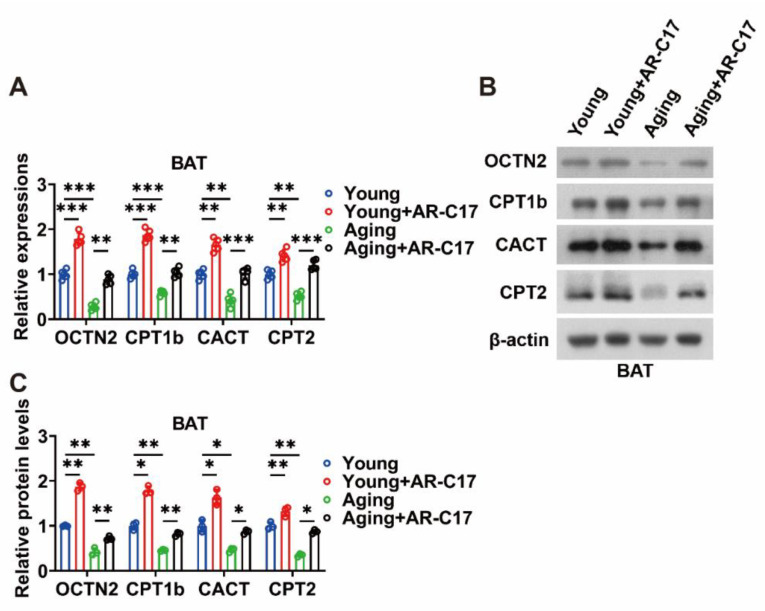
AR-C17 improves ACar metabolism in BAT of aging mice in response to cold. (**A**) Relative mRNA levels of organic cation transporter 2 (OCTN2), carnitine palmitoyltransferase 1b (CPT1a/b), carnitine palmitoyltransferase 2 (CPT2), and carnitine-acylcarnitine translocase (CACT) in BAT of young mice and aging mice treated with or without AR-C17 after 48 h cold exposure (*n* = 5). (**B**) Western blots of OCTN2, CPT1b, CACT, CPT2, and β-actin in BAT of young mice and aging mice treated with or without AR-C17 after 48 h cold exposure. (**C**) Relative protein levels of OCTN2, CPT1b, CACT, and CPT2 in BAT of young mice and aging mice treated with or without AR-C17 after 48 h cold exposure (*n* = 3). Data are presented as the mean ± SEM and *n* indicates the number of biologically independent experiments. Statistical significance was determined using a Two-tailed Student’s *t*-test. * *p* < 0.05; ** *p* < 0.01; *** *p* < 0.001; ns, not statistically significant.

The gene and protein expressions of the enzymes involved in the ACar metabolism of BAT were detected to clarify the effect of AR-C17 on the ACar metabolism of BAT. During cold exposure, the aging mice displayed lower gene expressions of OCTN2, CPT1b, CACT, and CPT2 in the BAT compared with the young mice, and AR-C17 could increase these gene expressions of the BAT in both young and aging mice (Figure 4A). That is to say, AR-C17 might reverse the aging-associated decline in gene expressions of the enzymes involved in the ACar metabolism of BAT. Furthermore, the protein expressions of OCTN2, CPT1b, CACT, and CPT2 in the BAT exhibited similar changes to the gene expressions. AR-C17 was found to increase these protein expressions of BAT in both the young and aging mice. Significantly, it also reversed the low protein expressions of the enzymes involved in the ACar metabolism of BAT in the aging mice (Figure 4B,C).

**Figure 5:**
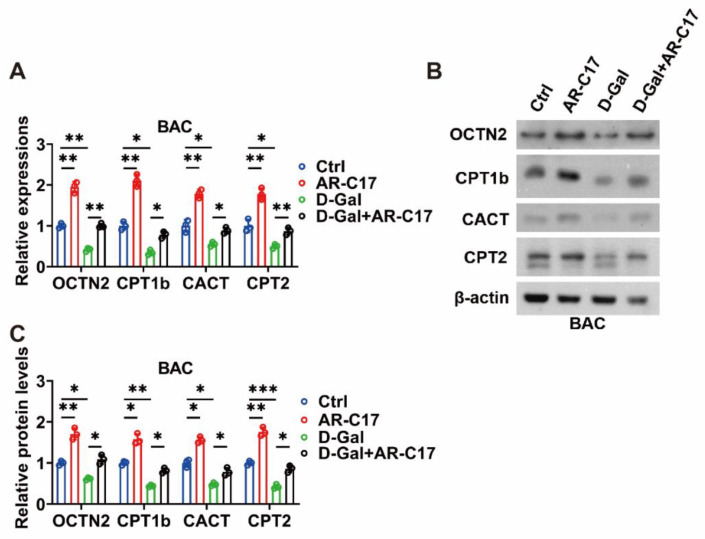
AR-C17 improves ACar metabolism of senescent BAC in vitro. (**A**) Relative mRNA levels of OCTN2, CPT1b, CACT, and CPT2 in D-galactose (D-Gal)-induced senescent BAC treated with or without AR-C17 (*n* = 3). (**B**) Western blots of OCTN2, CPT1b, CACT, CPT2, and β-actin in D-Gal-induced senescent BAC treated with or without AR-C17. (**C**) Relative protein levels of OCTN2, CPT1b, CACT, and CPT2 in D-Gal-induced senescent BAC treated with or without AR-C17 (*n* = 3). Data are presented as the mean ± SEM and *n* indicates the number of biologically independent experiments. Statistical significance was determined using a Two-tailed Student’s *t*-test. * *p* < 0.05; ** *p* < 0.01; *** *p* < 0.001; ns, not statistically significant.

Moreover, AR-C17 could increase the gene and protein expressions of the enzymes involved in the ACar metabolism of D-Gal-induced senescent BAC in vitro (Figure 5A–C). Therefore, our data suggested that AR-C17 might increase the ACar metabolism of BAT, particularly in aging mice, improving the transport and utilization of ACar in BAT and promoting BAT thermogenesis. Combined with the results of the ACar levels, it could be concluded that AR-C17 could increase the metabolic flux of ACar in BAT, thereby promoting the supply and utilization of ACar in the BAT of aging mice. Consequently, AR-C17 could improve the BAT thermogenesis of aging mice by promoting the ACar metabolism of BAT, and the ACar metabolism was involved in the regulatory network of AR-C17-mediated thermogenic regulation in aging mice.

### 3.4. AR-C17 Improves Glucose Levels and Lipid Profile of Aging Mice

**Figure 6:**
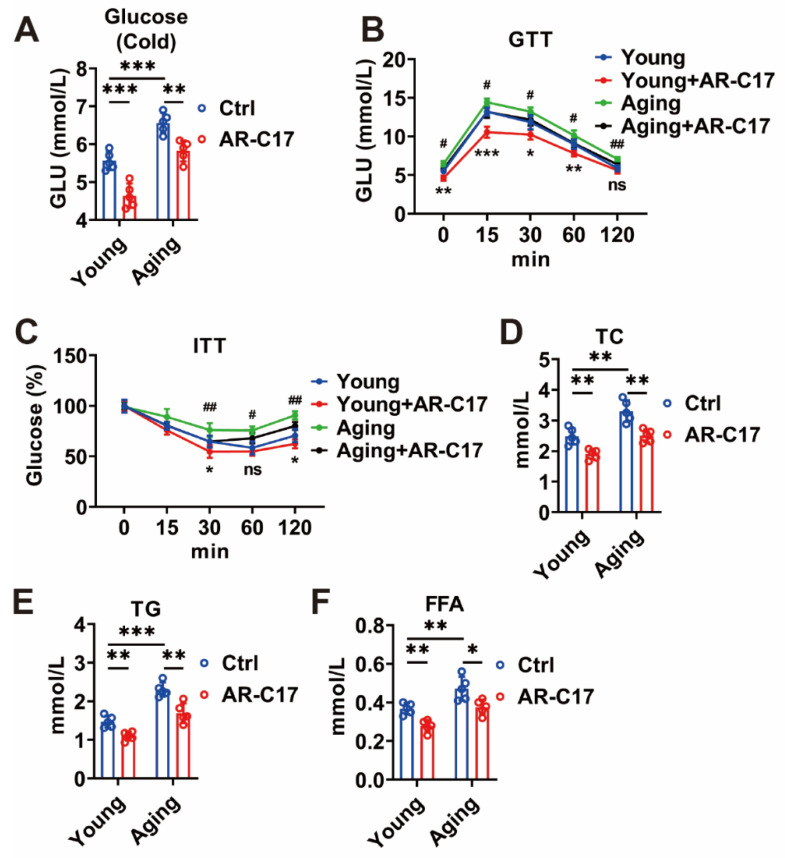
AR-C17 improves glucose levels and lipid profile of aging mice. (**A**) Blood glucose of young mice and aging mice treated with or without AR-C17 after 48 h cold exposure (*n* = 5). (**B**,**C**) Glucose tolerance test (GTT) (**B**) and insulin tolerance test (ITT) (**C**) of young mice and aging mice treated with or without AR-C17 (*n* = 5). * represents the significance between the young group and the young + AR-C17 group, and # represents the significance between the aging group and the aging + AR-C17 group. (**D**) Serum cholesterol (TC) level of young mice and aging mice treated with or without AR-C17 after 48 h cold exposure (*n* = 5). (**E**) Serum triglyceride (TG) level of young mice and aging mice treated with or without AR-C17 after 48 h cold exposure (*n* = 5). (**F**) Serum free fatty acid (FFA) level of young mice and aging mice treated with or without AR-C17 after 48 h cold exposure (*n* = 5). Data are presented as the mean ± SEM and *n* indicates the number of biologically independent experiments. Statistical significance was determined using a Two-tailed Student’s *t*-test. * *p* < 0.05; ** *p* < 0.01; *** *p* < 0.001; ns, not statistically significant. # *p* < 0.05; ## *p* < 0.01. In (**B**) and (**C**), * represents the significance between the young group and the young + AR-C17 group, and # represents the significance between the aging group and the aging + AR-C17 group.

Our previous study demonstrated that AR-C17 could increase the energy metabolism of aging mice [9]. Therefore, this study evaluated the glucose and lipid levels of young and aging mice treated with or without AR-C17. The findings demonstrated that AR-C17 effectively lowered the blood glucose levels in the aging mice exposed to cold conditions (Figure 6A). This suggests AR-C17 could enhance blood glucose clearance and ameliorate glucose levels in aging mice during cold exposure. Additionally, the GTT and ITT revealed impaired glucose tolerance and insulin sensitivity in the aging mice, which were improved by AR-C17 treatment (Figure 6B,C). Furthermore, the aging mice exhibited elevated serum levels of total TC, TG, and FFA compared to the young mice. AR-C17 supplementation effectively reduced these lipid markers in the aging mice (Figure 6D–F), mitigating lipid accumulation and improving the lipid profile of the aging mice. Consequently, AR-C17 improved the glucose levels and lipid profile and preserved the overall metabolic health of the aging mice. In conclusion, these benefits of AR-C17 in aging mice are likely linked, at least in part, to the enhancement of ACar metabolism in BAT.

## 4. Discussion

AR-C17 has been proven to improve the BAT thermogenesis of aging mice via regulating the Sirt3 signaling pathway, and Sirt3 may be a direct target of AR-C17 in the BAT of mice [9]. This study demonstrated that AR-C17 could regulate ACar metabolism in the BAT of mice. Therefore, there might be an interaction between Sirt3 and ACar metabolism. Sirt3 has been proven to mediate metabolic reprogramming by destabilizing hypoxia-inducible factor-1α (HIF1α) [30], and it was noticed that peroxisome proliferator-activated receptor alpha (PPARα) has been identified as a downstream target of HIF1α, and the activation of HIF1α inhibits the expression of PPARα [31]. Furthermore, PPARα has been proven to be the transcription factor of OCTN2, CPT1b, CACT, and CPT2 [32,33,34], regulating ACar metabolism at the transcriptional level. Therefore, Sirt3 may regulate ACar metabolism through the HIF1α-PPARα signaling pathway. Moreover, AR-C17 may promote ACar metabolism in the BAT of aging mice by regulating the Sirt3-HIF1α-PPARα signaling pathway, inducing the metabolic reprogramming of the BAT and improving the BAT thermogenesis of aging mice.

During cold exposure, plasma ACar, as a kind of thermogenic fuel, can be transported to BAT for the body’s thermogenesis to resist cold. Short-chain ACar can be transported into cells through the cell-surface transporter OCTN2 [35]. However, it is not established that OCTN2 can transport medium-chain ACar and long-chain ACar. The mechanism by which medium- and long-chain ACar are transported into cells is not understood. Therefore, the variations of OCTN2 in the BAT simply reflect the transportation of short-chain ACar from circulation to BAT. The transport of medium- and long-chain ACar from circulation to BAT can only be speculated based on the changes in the ACar levels in the plasma and BAT. However, it is important to note that the changes in the ACar levels in the BAT cannot exclude the effect of ACar utilization in BAT. Thus, it might be difficult to accurately evaluate the transport of medium- and long-chain ACar into cells. Therefore, future research should focus on investigating the precise transport mechanism of medium- and long-chain ACar from circulation to cells. Our study revealed that AR-C17 could promote the transport of short-chain ACar from circulation to BAT and might increase the metabolic flux of medium- and long-chain ACar in the BAT of aging mice, increasing the supply and utilization of thermogenic fuel in the BAT of aging mice during cold exposure.

AR-C17 has been demonstrated to regulate numerous metabolic actions. AR-C17 could alleviate oxidative damage and mitochondria-mediated apoptosis by regulating the Sirt3/FOXO3a signaling pathway in neurocytes, and AR-C17 could be a potential nutraceutical for neuroprotection [3]. AR-C17 could improve cognitive dysfunction and neuroinflammation, making it a potential dietary functional ingredient for preventing Alzheimer’s disease [4]. Furthermore, AR-C17 has been shown to have potential as a nutraceutical for managing breast cancer. AR-C17 could inhibit the proliferation of breast cancer cells by regulating the phosphoinositide 3-kinase (PI3K)/protein kinase B (AKT)/mammalian target of the rapamycin (mTOR) signaling pathway [5]. A previous study reported that AR-C17 could protect endothelial cells against arteriosclerosis by regulating the Sirt3 signaling pathway [6]. Furthermore, AR-C17 has been shown to have protective effects on adipocyte mitochondrial dysfunction by promoting Sirt3-mediated autophagy, and AR-C17 could be considered a potential dietary functional ingredient for preventing obesity [7]. Moreover, a recent study revealed that AR-C17 could prevent obesity and insulin resistance by activating BAT [8]. Our previous study suggested that AR-C17 could regulate BAT metabolism and improve the thermogenic capacity of aging mice by modulating the Sirt3-adenosine monophosphate-activated protein kinase (AMPK) signaling pathway, and AR-C17 might be a potential dietary functional ingredient to alleviate the decline in BAT function associated with aging [9]. Based on the previous study, it was found that AR-C17 could improve ACar metabolism in the BAT of aging mice, and ACar metabolism might be a deeper metabolic mechanism involved in the AR-C17-mediated thermogenic regulation of BAT.

## 5. Conclusions

**Figure 7:**
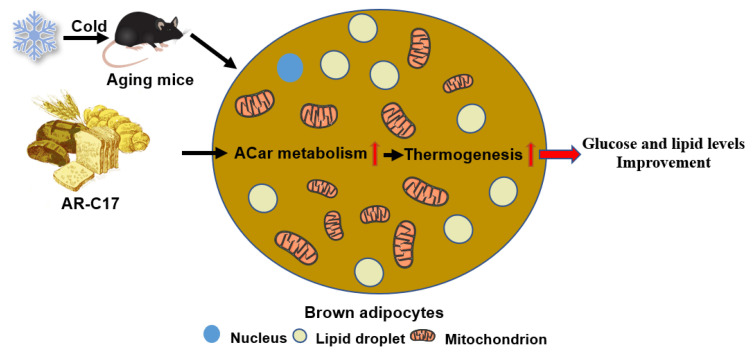
Schematic diagram of the working model in this study.

In this study, AR-C17 was found to promote ACar metabolism in the BAT of aging mice, resulting in improved BAT thermogenesis, decreased serum glucose levels, and improved lipid profile in the aging mice (Figure 7). This study revealed a deeper metabolic mechanism involved in the thermogenic regulation of BAT in aging mice mediated by AR-C17. It provides substantial evidence that AR-C17 could improve the decline in BAT function associated with aging, providing an important theoretical basis for WG’s nutrition and health benefits.

## Figures and Tables

**Table 1:** Chow composition.

Chow Composition	
Corn	Grain-based raw materials account for 80%
Wheat middlings	
Wheat	
Alfalfa	
Soybean meal	
Peruvian fish meal	Animal protein accounts for 10%
American chicken meal	
Animal premix	Small additive accounts for 10%
Gluten	
Calcium hydrogen phosphate	
Stone powder	
Salad-oil	
Feed-grade sodium chloride	
Feed-grade magnesium chloride	
Total	1
Total energy, kcal/kg	3616
Energy ratio of protein, %	20.6
Energy ratio of fat, %	12.0
Energy ratio of carbohydrate, %	67.4
Total	100%

## Data Availability

The data presented in this study are available upon request from the corresponding author.

## Supplementary Materials

The following supporting information can be downloaded at: https://www.mdpi.com/article/10.3390/nu15214597/s1, Table S1: Primer sequences. Table S2: RNA integrity. Supplementary Material MetaboAnalyst analysis.

## References

[1] Fan F., Zou Y., Fang Y., Li P., Xia J., Shen X., Liu Q., Hu Q. (2020). Potential neuroprotection of wheat alkylresorcinols in hippocampal neurons via Nrf2/ARE pathway. Food Funct..

[2] Jawhara M., Sorensen S.B., Heitmann B.L., Andersen V. (2019). Biomarkers of Whole-Grain and Cereal-Fiber Intake in Human Studies: A Systematic Review of the Available Evidence and Perspectives. Nutrients.

[3] Liu J., Wang Y., Hao Y., Wang Z., Yang Z., Wang Z., Wang J. (2020). 5-Heptadecylresorcinol attenuates oxidative damage and mitochondria-mediated apoptosis through activation of the SIRT3/FOXO3a signaling pathway in neurocytes. Food Funct..

[4] Liu J., Wang Y., Wang Z., Hao Y., Bai W., Wang Z., Wang J. (2020). 5-Heptadecylresorcinol, a Biomarker for Whole Grain Rye Consumption, Ameliorates Cognitive Impairments and Neuroinflammation in APP/PS1 Transgenic Mice. Mol. Nutr. Food Res..

[5] Xie M., Liu J., Wang Z., Sun B., Wang J. (2020). Inhibitory effects of 5-heptadecylresorcinol on the proliferation of human MCF-7 breast cancer cells through modulating PI3K/Akt/mTOR pathway. J. Funct. Food..

[6] Hao Y., Yang Z., Li Q., Wang Z., Liu J., Wang J. (2022). 5-Heptadecylresorcinol Protects against Atherosclerosis in Apolipoprotein E-Deficient Mice by Modulating SIRT3 Signaling: The Possible Beneficial Effects of Whole Grain Consumption. Mol. Nutr. Food Res..

[7] Hao Y., Yang Z., Liu J., Wang Z., Speakman J.R., Niu C., Sun B., Wang J. (2022). Protective effects of 5-heptadecylresorcinol against adipocyte mitochondrial dysfunction through upregulation of Sirt3-mediated autophagy. J. Nutr. Biochem..

[8] Yang Z., Yang S., Wang Z., Hao Y., Wang Z., Wei Y., Ye G., Liu J., Wang J. (2023). 5-Heptadecylresorcinol alleviated high-fat diet induced obesity and insulin resistance by activating brown adipose tissue. Food Funct..

[9] Zhang K., Li T., Li Q., Nie C., Sun Y., Xue L., Wang Y., Fan M., Qian H., Li Y. (2023). 5-Heptadecylresorcinol Regulates the Metabolism of Thermogenic Fat and Improves the Thermogenic Capacity of Aging Mice via a Sirtuin 3-Adenosine Monophosphate-Activated Protein Kinase Pathway. J. Agric. Food Chem..

[10] Gong L., Cao W., Chi H., Wang J., Zhang H., Liu J., Sun B. (2018). Whole cereal grains and potential health effects: Involvement of the gut microbiota. Food Res. Int..

[11] Zhang K., Sun J., Fan M., Qian H., Ying H., Li Y., Wang L. (2021). Functional ingredients present in whole-grain foods as therapeutic tools to counteract obesity: Effects on brown and white adipose tissues. Trends Food. Sci. Technol..

[12] Cohen P., Kajimura S. (2021). The cellular and functional complexity of thermogenic fat. Nat. Rev. Mol. Cell Biol..

[13] Villanueva-Carmona T., Cedo L., Madeira A., Ceperuelo-Mallafre V., Rodriguez-Pena M.M., Nunez-Roa C., Maymo-Masip E., Repolles-de-Dalmau M., Badia J., Keiran N. (2023). SUCNR1 signaling in adipocytes controls energy metabolism by modulating circadian clock and leptin expression. Cell Metab..

[14] Yan S., Zhou X., Wu C., Gao Y., Qian Y., Hou J., Xie R., Han B., Chen Z., Wei S. (2023). Adipocyte YTH N(6)-methyladenosine RNA-binding protein 1 protects against obesity by promoting white adipose tissue beiging in male mice. Nat. Commun..

[15] Xiao F., Jiang H., Li Z., Jiang X., Chen S., Niu Y., Yin H., Shu Y., Peng B., Lu W. (2023). Reduced hepatic bradykinin degradation accounts for cold-induced BAT thermogenesis and WAT browning in male mice. Nat. Commun..

[16] Wu J., Bostrom P., Sparks L.M., Ye L., Choi J.H., Giang A.H., Khandekar M., Virtanen K.A., Nuutila P., Schaart G. (2012). Beige adipocytes are a distinct type of thermogenic fat cell in mouse and human. Cell.

[17] Betz M.J., Enerback S. (2018). Targeting thermogenesis in brown fat and muscle to treat obesity and metabolic disease. Nat. Rev. Endocrinol..

[18] Wang X., Liu S.-Y., Hu G.-S., Wang H.-Y., Zhang G.-L., Cen X., Xiang S.-T., Liu W., Li P., Ye H. (2022). DDB1 prepares brown adipocytes for cold-induced thermogenesis. Life Metab..

[19] Simcox J., Geoghegan G., Maschek J.A., Bensard C.L., Pasquali M., Miao R., Lee S., Jiang L., Huck I., Kershaw E.E. (2017). Global Analysis of Plasma Lipids Identifies Liver-Derived Acylcarnitines as a Fuel Source for Brown Fat Thermogenesis. Cell Metab..

[20] Verkerke A.R.P., Kajimura S. (2021). Oil does more than light the lamp: The multifaceted role of lipids in thermogenic fat. Dev. Cell.

[21] Gnad T., Navarro G., Lahesmaa M., Reverte-Salisa L., Copperi F., Cordomi A., Naumann J., Hochhauser A., Haufs-Brusberg S., Wenzel D. (2020). Adenosine/A2B Receptor Signaling Ameliorates the Effects of Aging and Counteracts Obesity. Cell Metab..

[22] Wang G., Song A., Bae M., Wang Q.A. (2022). Adipose Tissue Plasticity in Aging. Compr. Physiol..

[23] Gohlke S., Zagoriy V., Cuadros Inostroza A., Meret M., Mancini C., Japtok L., Schumacher F., Kuhlow D., Graja A., Stephanowitz H. (2019). Identification of functional lipid metabolism biomarkers of brown adipose tissue aging. Mol. Metab..

[24] Cypess A.M., Weiner L.S., Roberts-Toler C., Franquet Elia E., Kessler S.H., Kahn P.A., English J., Chatman K., Trauger S.A., Doria A. (2015). Activation of human brown adipose tissue by a beta3-adrenergic receptor agonist. Cell Metab..

[25] Wang X., Xu M., Li Y. (2022). Adipose Tissue Aging and Metabolic Disorder, and the Impact of Nutritional Interventions. Nutrients.

[26] Nemkov T., Cendali F., Stefanoni D., Martinez J.L., Hansen K.C., San-Millan I., D’Alessandro A. (2023). Metabolic Signatures of Performance in Elite World Tour Professional Male Cyclists. Sports Med..

[27] Liu J., Nie C., Xue L., Yan Y., Liu S., Sun J., Fan M., Qian H., Ying H., Wang L. (2021). Growth hormone receptor disrupts glucose homeostasis via promoting and stabilizing retinol binding protein 4. Theranostics.

[28] Liu S., Shen S., Yan Y., Sun C., Lu Z., Feng H., Ma Y., Tang Z., Yu J., Wu Y. (2022). Triiodothyronine (T3) promotes brown fat hyperplasia via thyroid hormone receptor alpha mediated adipocyte progenitor cell proliferation. Nat. Commun..

[29] Li Y., Jiang J., Liu W., Wang H., Zhao L., Liu S., Li P., Zhang S., Sun C., Wu Y. (2018). microRNA-378 promotes autophagy and inhibits apoptosis in skeletal muscle. Proc. Natl. Acad. Sci. USA.

[30] Finley L.W., Carracedo A., Lee J., Souza A., Egia A., Zhang J., Teruya-Feldstein J., Moreira P.I., Cardoso S.M., Clish C.B. (2011). SIRT3 opposes reprogramming of cancer cell metabolism through HIF1alpha destabilization. Cancer Cell.

[31] Xu P., Chen C., Zhang Y., Dzieciatkowska M., Brown B.C., Zhang W., Xie T., Abdulmalik O., Song A., Tong C. (2022). Erythrocyte transglutaminase-2 combats hypoxia and chronic kidney disease by promoting oxygen delivery and carnitine homeostasis. Cell Metab..

[32] Rolver M.G., Holland L.K.K., Ponniah M., Prasad N.S., Yao J., Schnipper J., Kramer S., Elingaard-Larsen L., Pedraz-Cuesta E., Liu B. (2023). Chronic acidosis rewires cancer cell metabolism through PPARalpha signaling. Int. J. Cancer.

[33] Wen G., Ringseis R., Eder K. (2010). Mouse OCTN2 is directly regulated by peroxisome proliferator-activated receptor alpha (PPARalpha) via a PPRE located in the first intron. Biochem. Pharmacol..

[34] Zhao Y., Ling S., Li J., Zhong G., Du R., Li Y., Wang Y., Liu C., Jin X., Liu W. (2021). 3’ untranslated region of Ckip-1 inhibits cardiac hypertrophy independently of its cognate protein. Eur. Heart J..

[35] Wang A.G., Diamond M., Waddell J., McKenna M.C. (2019). Effect of Acetyl-L-carnitine Used for Protection of Neonatal Hypoxic-Ischemic Brain Injury on Acute Kidney Changes in Male and Female Rats. Neurochem. Res..

