# Supplementary material for: The Enhancement of Acylcarnitine Metabolism by 5-Heptadecylresorcinol in Brown Adipose Tissue Contributes to Improving Glucose and Lipid Levels in Aging Male Mice
Source: Nutrients. 2023 Oct 29;15(21):4597. doi: 10.3390/nu15214597 (PMC10649465; doi:10.3390/nu15214597)
Supplement: Supplementary file 1 [file nutrients-15-04597-s001.zip › Supplementary Material MetaboAnalyst analysis.pdf]

Data normalization

In the “Data normalization” page, select “Normalization by sum” for sample normalization, “None” for data transformation and “Auto scaling” for data scaling. After selecting these options, click the “Normalize” button. Click “View Result” to view a graphical summary of the effect of data normalization on the data. The top plots show the overall data distribution based on kernel density estimation, while the two horizontal box plots on the bottom show the distributions of individual variables or metabolite concentrations. Users should compare the graphical summary on the left and the right to guide them toward choosing the methods that work best with their data. Once a user is satisfied with the shape and skewness of the curve (looking like a symmetric Gaussian curve), click the “Proceed” button. Prior to conducting any kind of data analysis in metabolomics, it is important to assess the overall data quality and check for any obvious outliers. As there are no obvious outliers based on the above procedures, we will redo our data normalization to create some “artificial” outliers—for illustration purposes only. Click the “Normalization” hyperlink on the navigation tree to return to the “Data Normalization” page. Make sure to set the Sample normalization to “none,” choose “Auto scaling” for Data scaling, and click the “Normalize” button. The previously normalized data is now overwritten by the new data containing several artificial outliers. At this point, the “Data Normalization Result” page should be displayed. Click the “Proceed” button to move on to the “Data Analysis Overview” page.

PCA

In the “Data Analysis Overview” page, Click the “principal component analysis” hyperlink on the main page or the “PCA” hyperlink on the left navigation tree. After a few seconds, the PCA results should be presented in a multi-panel page. The default panel shows a pair-wise scores plot between the first five principal components (PCs). The variance explained by each PC is shown on the corresponding diagonal cell. As the first three PCs account for similar levels of variances in the data, they will be the focus of the remainder of the analysis. Change the number of displayed PCs from 5 to 3, and then click the “Update” button. These data points are drawn in different colors and shapes based on their group memberships. Click the “2D Score Plot” tab to get a more detailed score plot. Click the “Loadings Plot” tab to view the PCA loadings plot. Click the “Synchronized 3D Plots” to further explore the PCA results in an interactive 3D score and loading plot of the first three PCs.

PLSDA

To perform PLS-DA analysis, click the “PLSDA” hyperlink on the navigation tree. Wait for about10 sec for MetaboAnalyst to finish its default analysis. Like PCA, the

results are presented in a multi-panel page with the pair-wise score plots of the first five components shown as a default. Click the “2D Scores Plot” tab at the top of the page to view the scores plot between the first two components. A much better separation is obtained compared to PCA. Click the “Loadings Plot” tab, and the result is shown. Click the outermost points along the directions of separation to identify the most influential metabolites. Most of these metabolites agree very well with those metabolites already identified by both PCA and Heatmap techniques. PLSDA is a supervised classification method. It uses the group label to maximize the separation between different groups. One important issue associated with PLSDA is overfitting. To address this issue, MetaboAnalyst provide two approaches—cross validation and permutation testing. Click the “Cross Validation” tab to view the results from cross validation. The purpose of cross validation is to determine the optimal number of components needed to build the PLS-DA model. There are three common performance measures—the sum of squares captured by the model (R^2), the cross-validated R^2 (also known as Q^2), and the prediction accuracy (Accuracy). Select R^2 as the performance measure. Click the “Permutation” tab to see the results from MetaboAnalyst’s permutation tests. There are two parameters for permutation tests—the number of permutations and the test statistic that will be used as a performance measure. MetaboAnalyst provides two options for its test statistics: (a) the separation distance which is defined as the ratio of the between-group sum of the squares and the within-group sum of squares (B/W-ratio) or (b) the prediction accuracy. Click the “Imp. Features” tab to view the most important or informative metabolites that were selected using the three-component model. The default view shows the top 25 compounds ranked based on the variable importance in projection (VIP) score.
